# Rypture of an Umbilical Hernia

**Published:** 1858-12

**Authors:** 


*Rupture of an Umbilical Hernia, and escape of a large mass of Intestines—Peritonitis—Opium—Recovery.* By Reed B. Bontecou, M. D. Troy, N. Y.

*August 9th*, 1857—I was called to see Mrs. W. at 10" P. M. Found the patient (a stout, healthy looking woman,) lying in bed with her clothes on, her countenance indicating terrible anxiety and suffering. She implored me to do something for her, saying that she had burst; that she was six months pregnant, and that a physician who came to see her about five o'clock, soon after the accident, had pronounced her case as hopeless. I laid bare the abdomen, and found a large mass of intestines and omentum, much swollen and very red, lying by her side on a coarse flannel skirt. They had escaped from the vicinity of the umbilicus, and made a tumor large enough to fill a peck measure. It consisted of nearly all the smaller intestines, with omentum; was covered with gravel adhering to the surface, and felt cold. I administered a grain of morphine immediately, and cleaned the protruding mass as well as the convenience at hand would allow. On making an attempt to reduce it, I found it so swollen and œdematous as to preclude the possibility of doing so without enlarging the aperture, which I did at once with a probe-pointed bistoury, cutting freely upwards in the mesian line, and returning the whole without further difficulty.

Constant efforts at vomiting made it difficult to retain the parts until sutures were introduced. I pinched firmly together at its base a sac the size of an ordinary orange, including the umbilicus and a rent through which the parts had escaped, and placed four interrupted sutures through it. The flabby sac I then rolled up on itself and made answer the purpose of a compress, keeping it in its place by another compress, a girdle of adhesive plaster, and a bandage of cloth. The uterine tumor was of course apparent, and motion of the fœtus was felt. She had been laboring under an umbilieal hernia since the birth of her youngest child two years previous, and during the present pregnancy it had increased in size, at times as large as the fist, and the skin covering the sac had for some time previous been tender, as if an abscess was forming in it. The accident occurred to her while stooping to feed her chickens, at about 5 P. M.; the parts had therefore been protruded five hours when I first saw her.

The pulse was small and frequent, and she was still suffering from the shock of the accident. I gave her ten grains of opium in powder, and left others with directions to give one every two hours till she slept. The frequent large doses were given in anticipation of peritonitis, which I thought inevitable.

*August* 10*th*, 8½ *A.M.*—The patient had not slept, and the powders had been given according to directions, making in all 50 grs. besides the morphine. She was lying on her back, the limbs flexed, breathing easily, and quite free from pain. The vomiting had ceased soon after I left on the previous evening. The pupils were contracted, eyes bright, no drowsiness, tongue moist; was rational, and much terrified with the apprehension of death; had not passed urine, but made no complaint of distress on that account; could not detect any distension of the bladder. I left a number of ten gr. powders of opium; ordered them continued every two hours as before; prohibited much nourishment or drink.

3 *P. M.*—Is much the same as in the morning; complained of some pain in the lower part of abdomen, and had felt unusual motion of the child; had had no sleep, but attributed her wakefulness to the excitement of many persons running in to see her. She appeared rational, pupils contracted, eyes bright, tongue moist, no vomiting, no great thirst, and no desire for nourishment; pulse 90 and rather small; ordered the ten gr. opium powders continued every two hours as before.

9 *P. M.*—Patient still wakeful and complaining of pain all over the lower part of the abdomen. She had not passed urine since the accident, and I evacuated it with the catheter. Pulse 100, pupils contracted, eyes bright, tongue moist, with thin pale fur, skin moist. Prescribed beef tea as nourishment, and 15 grs. pulv. opium every two hours till pain was subdued and sleep obtained. She was rational and composed.

11*th*, 9 *A. M.*—Patient still wakeful, quite easy, rational, and inclined to be cheerful. Since 5 A. M. had been vomiting a clear watery fluid without nausea. The powders had been given as prescribed, and had not been rejected. She was somewhat annoyed with motion of fœtus. Had her clothes changed for the first time since the accident; pulse about 90; in other respects in much the same condition as on the previous evening. Prescribed ½ minim of creasote in bread-crumb pill for the vomiting, and to continue the 15 gr. powder of opium every two hours.

8 *P. M.*—Patient had not yet slept, though the house had been kept quiet; was quite comfortable, having suffered little pain except in the wound: was unable to extend the limbs; the lower part of the abdomen distended and tympanitic; had passed urine, ordered hot fomentations sprinkled with spts. turpentine, to lower part of the bowels, and a dose of oil and turpentine internally as a laxative. Prescribed ten grs. opium every three hours till sleep was obtained, and oftener if in much pain.

12*th*, 10 *A. M.*—Patient had slept 3 hours, and was sweating profusely; had passed some urine, but the bowels had not moved; the abdomen was greatly distended and tympanitic, causing pain in the wound and over the belly generally. I loosened the bandage and adhesive girth. She spoke of having felt much motion of the fœtus. Ordered oat meal gruel and beef tea as nourishment. Prescribed salts ℥ss. to be taken at once, and to use an enema in four hours; to continue the ten-grain opium powder, and use the fomentations as before; the vomiting had ceased.

Before evening her husband came for me, saying she was terribly bloated and suffering more pain, notwithstanding the powders had been given every two hours. I saw her about 7 P. M. She had had three profuse, thin, watery evacuations from the bowels, and was much relieved of the distension and pain. She was rational and bright; skin moist, pupils contracted, pulse 100.

13*th*, 9 *A. M.*—Patient sleeping; soon awoke and conversed cheerfully, expressing much gratitude for my services; had slept much during the night; was quite free from pain, and could extend the limbs a little; very tired of the bed, and wanted permission to get up; pulse 90; skin moist; eyes bright; tongue coated, but moist; some appetite; allowed roast oysters, and prescribed 5 grs. opium in powder every four hours.

14*th*, 10 *A. M.*—Had slept most of the night; felt well, except pain and soreness in the wound; pulse 82. I dressed the wound for the first time, now five days since the operation. Found considerable swelling of the parts about the sutures, and withdrew them. The walls of the sac were apparently adherent, and I left it rolled up on itself as it had been; covered the whole with lint, spread with simple cerate, and an adhesive girth about the body. Prescribed a continuance of the 5 gr. powders of opium every four or five hours. She continued to improve, till twelve days from the time of the accident, when she aborted *at* or *about* the sixth month. I continued to administer opium, 15 or 20 grs. daily, occasionally combining with it acetat. plumbi or tannin, or both, to correct a tendency to diarrhœa, which annoyed her after the miscarriage. I continued my visits *daily* till the 1st of September; after that *occasionally* for three weeks, during which time I gave opium largely. It was some weeks before she could extend her limbs sufficiently to stand erect.

This person has since given birth to a healthy child, and is herself robust. The hernia, I may mention, never appeared after the operation.

---

*Statistics of Tracheotomy.*—The statistics of the operations of tracheotomy performed during a number of years at the *Hôpital des Enfans* at Paris, where the effects can be observed upon an extended scale, must always be interesting and valuable. In former years we have frequently entered into practical details on the subject. We now quote from the *Journal of Practical Medicine and Surgery* the following statistics relative to the operations of tracheotomy performed during the eight years just elapsed.

The following is the list of these operations from 1850 through 1857, with the number of cures obtained:

| | | | |
|---|---|---|---|
| 1850—20 | operations | 6 | recoveries. |
| 1851—31 | " | 12 | " |
| 1852—59 | " | 11 | " |
| 1853—61 | " | 7 | " |
| 1854—45 | " | 11 | " |
| 1855—48 | " | 10 | " |
| 1856—55 | " | 14 | " |
| 1857—71 | " | 15 | " |
| Total, 390 | | 86 | |

It will be seen by the above table, that the proportion of recoveries, although very unequal in the several years, presents a very similar general average; that is, from 1 in 4 to 1 in 5 of the whole number operated on yearly. It should be mentioned that the majority of the children operated on were in the last stage of croup, and were consequently in imminent danger of death.

M. Guersant, in whose wards this estimate was prepared, gives the following summary of the indications for and against tracheotomy, based upon the age of the children, for the existing complications, &c.

Age is an important element to be considered. Amongst the cases which compose the above table, there is one of a child 18 months old, who died with convulsions during tracheotomy. M. Chailon, the author of the article cited by us from the *Journal of Practical Medicine and Surgery*, states that he saw, on the 7th of January last, a little girl of two and a half years die during the operation, notwithstanding the well-known skill of the surgeon. He had also seen a similar case in private practice—the patient being also a girl less than three years old.

Nevertheless, whilst the peculiar difficulties of tracheotomy in subjects under the age of two years are admitted—difficulties ascribable to the restricted relations and volume of the parts at that age; to the dangers of a minute, long and delicate dissection; and especially to the small size and mobility of the trachea, which often allow the insertion of the tube only with extreme difficulty—M. Guersant does not consider the youth of the patient an absolute contra-indication to tracheotomy.

The same is true as regards pneumonia, when it complicates pseudo-membranous croup. For a long time, says M. Chailon, the existence of this complication was thought sufficient wholly to contra-indicate tracheotomy. At present, M. Guersant adopts the opposite opinion; and he has become convinced that, in establishing respiration by an artificial track, he has favored the resolution of the pneumonia. He admits but one decided contra-indication to opening the trachea in croup—and that is, diphtheritic infection, or general diphtheritis. When a child whose vocal chords have been invaded by false membranes, exhibits at the same time similiar morbid products in the nose, the ears, or upon the skin; when there are attacks of epistaxis and every sign of extreme debility—tracheotomy will be useless; the child will invariably die.

M. Guersant does not, moreover, consider the extremest degree of asphyxia an insurmountable obstacle to the success of the operation, provided the condition is permanent, and has continued for at least an hour, with a persistent character.

Slow and continued asphyxia is, indeed, the very state which is the chief indication for tracheotomy, according to M. Guersant. It is then the only thing to be done—the reestablishment of respiration being that alone which can keep the child alive.

There is a sort of asphyxia which does not so imperatively call for the operation—viz., the intermittent form. M. Guersant has seen children making violent efforts to breathe and seemingly about to die instantly; false membrane having been discharged, the nature of the disease was certain. Notwithstanding, the friends having opposed the operation deemed necessary by the surgeon, the usual means were employed—such as emetics, calomel, alum, and chlorate of potash—and the patients have recovered. But with the exception of these rare instances and of the far more common cases of general diphtheritis, M. Guersant thinks that as a general prin[illegible]iple, tracheotomy is distinctly indicated whenever there is con[illegible]inued and increased embarrassment of the respiration.—[*Gazette des Hôpitaux, and Boston Med. and Surg. Journal.*

